# TET1 contributes to allergic airway inflammation and regulates interferon and aryl hydrocarbon receptor signaling pathways in bronchial epithelial cells

**DOI:** 10.1038/s41598-019-43767-6

**Published:** 2019-05-14

**Authors:** J. D. Burleson, Dylan Siniard, Veda K. Yadagiri, Xiaoting Chen, Matthew T. Weirauch, Brandy P. Ruff, Eric B. Brandt, Gurjit K. Khurana Hershey, Hong Ji

**Affiliations:** 1 Division of Asthma Research, Cincinnati Children’s Hospital Medical Center, Cincinnati, OH USA; 2 Pyrosequencing lab for genomic and epigenomic research, Cincinnati Children’s Hospital Medical Center, Cincinnati, OH USA; 3 Center for Autoimmune Genomics and Etiology, Cincinnati Children’s Hospital Medical Center, Cincinnati, OH USA; 4 Divisions of Biomedical Informatics and Developmental Biology, Cincinnati Children’s Hospital Medical Center, Cincinnati, OH USA; 5 Department of Pediatrics, University of Cincinnati College of Medicine, Cincinnati, OH USA; 6 Department of Anatomy, Physiology and Cell Biology, School of Veterinary Medicine, University of California, Davis, CA USA; 7 California National Primate Research Center, Davis, CA USA

**Keywords:** Asthma, DNA methylation

## Abstract

Previous studies have suggested a role for Tet1 in the pathogenesis of childhood asthma. However, how Tet1 contributes to asthma remains unknown. Here we used mice deficient for Tet1 in a well-established model of allergic airway inflammation and demonstrated that loss of Tet1 increased disease severity including airway hyperresponsiveness and lung eosinophilia. Increased expression of *Muc5ac*, *Il13*, *Il33*, *Il17a*, *Egfr*, and *Tff*2 were observed in HDM-challenged Tet1-deficient mice compared to Tet1^+/+^ littermates. Further, transcriptomic analysis of lung RNA followed by pathway and protein network analysis showed that the IFN signaling pathway was significantly upregulated and the aryl hydrocarbon receptor (AhR) pathway was significantly downregulated in HDM-challenged Tet1^−/−^ mice. This transcriptional regulation of the IFN and AhR pathways by Tet1 was also present in human bronchial epithelial cells at base line and following HDM challenges. Genes in these pathways were further associated with changes in DNA methylation, predicted binding of transcriptional factors with relevant functions in their promoters, and the presence of histone marks generated by histone enzymes that are known to interact with Tet1. Collectively, our data suggest that Tet1 inhibits HDM-induced allergic airway inflammation by direct regulation of the IFN and AhR pathways.

## Introduction

Asthma is one of the most common chronic disorders in childhood^1^, currently affecting an estimated 6.2 million children under 18 years, of which 3.1 million suffered from an asthma attack or episode in 2015^2^. The annual cost of asthma is estimated to be $50.1 billion, representing $3,100 per person per year, more than half of which is related to prescription medication costs^3–5^. Although multiple asthma therapies exist, the heterogeneous nature of asthma and great variability among patients’ individual therapeutic responses highlight the need for alternative individualized therapies based on a better understanding of disease mechanisms^6,7^. A growing body of epidemiological studies, including ours^8–10^, have identified epigenetic variations associated with asthma and asthma severity in various tissues and suggested an important role for DNA methylation (DNAm) in asthma pathogenesis^8–13^. Studies in murine models demonstrated the role of DNA methylation in T cell polarization and cytokine production^14,15^, and that pharmacologic inhibition of DNA methylation in CD4^+^ T cells protected against the development of allergic airway inflammation^15^. Studies of lung tissues from a mouse model of experimental asthma identified DNA methylation changes related to smooth muscle functions in mice with allergic asthma^16,17^. However, further mechanistic studies are needed to understand the pathways through which DNA methylation contribute to asthma features, especially in the regulation of the airway epithelium, a major player in asthma due to its unique interface with the environment and interaction with immune cells^18–20^.

Our previous publications have associated *TET1* promoter methylation with childhood asthma^8,9^. Tet1 is known to regulate gene expression in many cell types including embryonic stem cells (ESC) and HEK293 cells^21–25^. Tet1 belongs to the mammalian demethylase family (TET1/2/3) that hydrolyzes 5-methyl-cytosine (5mC) to generate 5hmC, usually resulting in activation of gene expression^26–30^. In addition to its catalytic activity, the TET1 protein recruits histone modifying protein complexes (e.g. OGT/SET1, SIN3A/HDAC and EZH2/SUZ12/EED) to alter histone marks and chromatin accessibility, leading to both activation and repression of gene expression^31–34^. TET1 is involved in many biological processes and diseases, including stem cell maintenance, T and B cell development, genomic imprinting, neural activity and cancer^35–43^. The expression of *TET1* is regulated by asthma-related exposures such as diesel exhaust particles and house dust mite (HDM) in human bronchial epithelial cells^8,9^ and in whole lungs of mice^16^. However, the contributions of *TET1* to asthma remain unknown.

**Figure 1:**
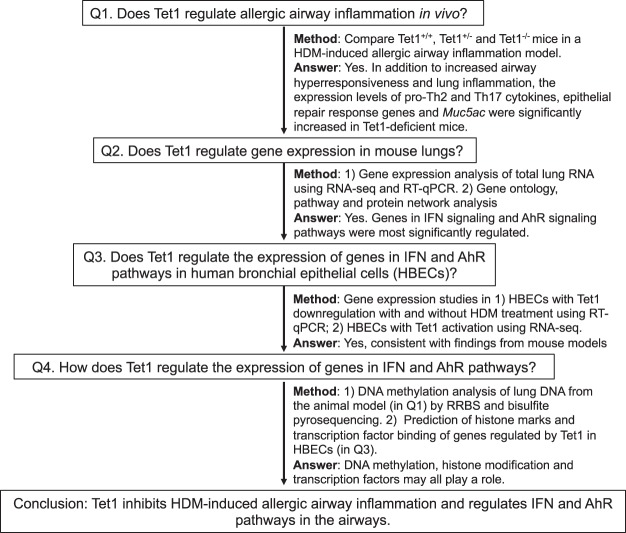
Summary of study design.

In this paper, we examined the role of Tet1 in allergic airway inflammation using animal models and cell lines (Fig. 1, study design). We applied integrative transcriptomic analysis, pathway and protein interaction network analysis to identify genes and pathways regulated by Tet1 in HDM-exposed murine lungs and in human bronchial epithelial cells. We then examined the specific regulation of candidate genes in identified pathways by TET1 in bronchial epithelial cells at base line and following acute HDM challenges. Finally, we performed DNA methylation studies and functional genomic analyses to understand how Tet1 regulates gene expression in the airways. Collectively, our data from both mouse models and human bronchial epithelial cells strongly support that Tet1 suppresses allergic airway inflammation by transcriptional regulation of Interferon (IFN) and Aryl hydrocarbon Receptor (AhR) signaling pathways through changes in DNA methylation, and interactions with particular transcription factors and histone modifiers.

## Results

### Loss of Tet1 exacerbates HDM-induced AHR and lung eosinophilia

**Figure 2:**
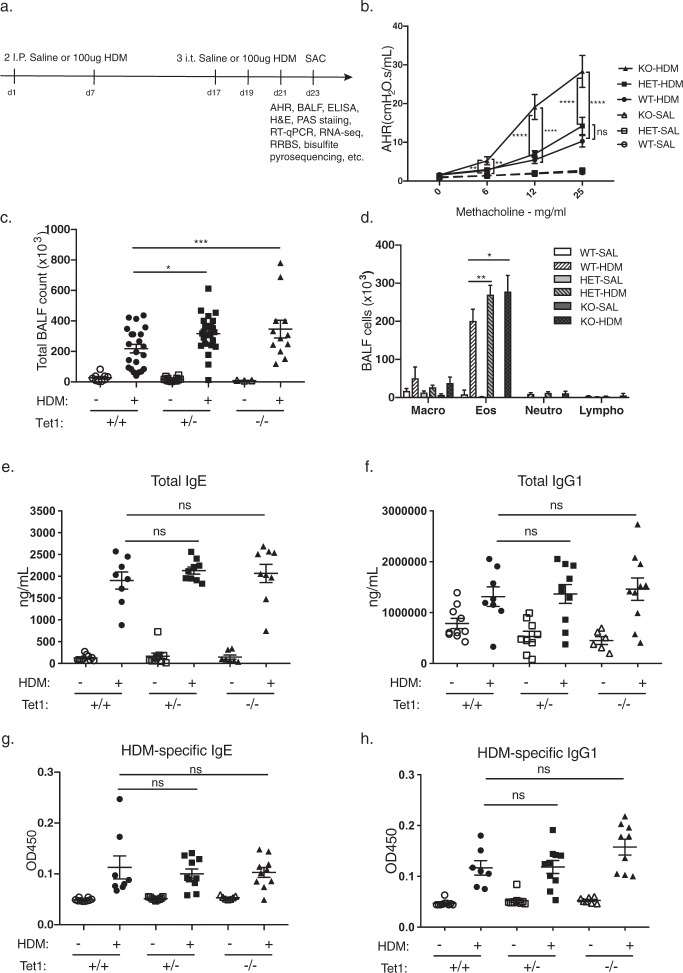
Loss of Tet1 exacerbated airway hyperresponsiveness and lung inflammation in a mouse model of asthma. (**a**) Treatment protocol. (**b**) Airway hyperresponsiveness. (**c**) Total BALF cells. (**d**) Number of each cell type. (**e**) Total IgE. (**f**) Total IgG1. (**g**) HDM-specific IgE (1:5 dilution). (**h**) HDM-specific IgG1 (1:2000 dilution). Tet1^+/+^ Saline, n = 8–15; Tet1^+/+^ HDM, n = 7–24; Tet1^+/−^ Saline, n = 9–14; Tet1^+/−^ HDM, n = 9–24; Tet1^−/−^ Saline, n = 6–7; Tet1^−/−^ HDM, n = 9–16. mean ± SEM for each group is shown. Unpaired student t-test was applied with Bonferroni corrections for multiple testing. *p < 0.05, **p < 0.01, ***p < 0.001, ****p < 0.0001.

To determine the role of Tet1 in asthma development, we utilized an experimental model of allergic airway inflammation that was previously established^17,44^ (Fig. 2a). Loss of Tet1 significantly exacerbated HDM-induced AHR at methacholine doses of 6, 12 and 25 mg/ml (Fig. 2b). No significant increase in AHR was observed in the HDM-treated Tet1^+/−^ mice compared to HDM-treated Tet1^+/+^ mice (Fig. 2b). There were significantly more Bronchoalveolar lavage fluid **(**BALF) cells in both the HDM-challenged Tet1^+/−^ and Tet1^−/−^ mice (Fig. 2c). Significantly more eosinophils were observed in the BALF of Tet1^−/−^ and Tet1^+/−^ mice compared to Tet1^+/+^ mice (Fig. 2d), which may be driven by increased total cell number in BALF. In summary, these data suggest that loss of Tet1 increases HDM-induced AHR and lung inflammation. Sensitization was not significantly affected as exposure to HDM did not significantly alter total IgE, total IgG1, HDM-specific IgE and HDM-specific IgG1 levels in serum from Tet1^−/−^ and Tet1^+/−^ mice when compared to Tet1^+/+^ mice (Fig. 2e–h).

### Loss of Tet1 promotes the expression of pro-Th2 and Th17 cytokines and epithelial repair genes induced by HDM

**Figure 3:**
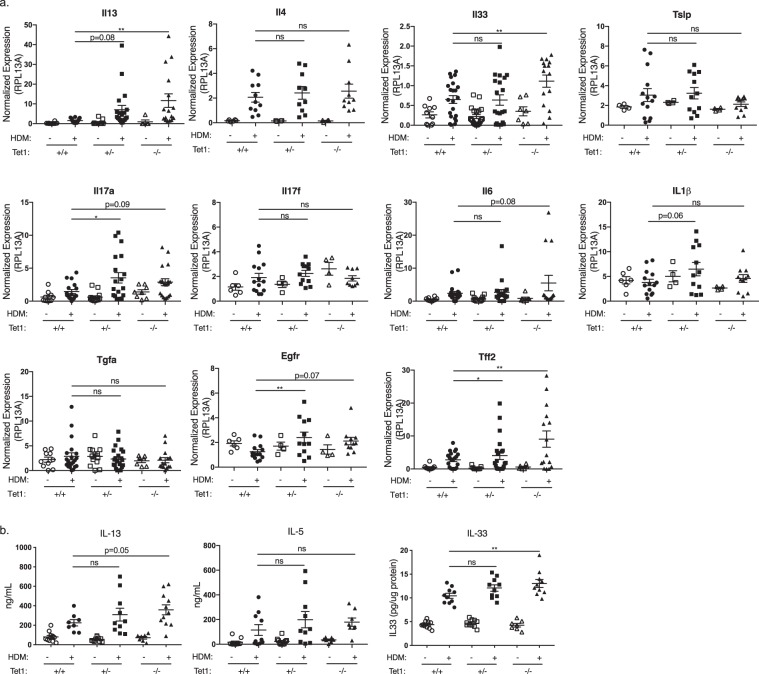
Tet1 deficiency increased the expression of pro-Th2/Th17 and epithelial repair response genes. (**a**) Expression levels of indicated genes were measured by RT-qPCR 48 hrs after the last i.t. HDM challenges. Expression values were normalized to the expression of mRpl13a. Mean ± SEM for each group is shown. Tet1^+/+^ Saline, n = 4–10; Tet1^+/+^ HDM, n = 11–22; Tet1^+/−^ Saline, n = 4–14; Tet1^+/−^ HDM, n = 12–21; Tet1^−/−^ Saline, n = 4–7; Tet1^−/−^ HDM, n = 10–19. Student t-tests were applied to analyze normally distributed data for *Il4*, *Il1b, Tslp, Il17f* and *Egfr* with Bonferroni corrections for multiple testing. For comparisons involving data that are not normally distributed in all other genes, Mann Whitney tests were applied. (**b**) Protein levels of IL13, IL5 and IL33 measured by ELISA. IL13 and IL5 were measured in BALF, and IL33 was measured in lung homogenate. Tet1^+/+^ Saline, n = 10–11; Tet1^+/+^ HDM, n = 8–10; Tet1^+/−^ Saline, n = 10; Tet1^+/−^ HDM, n = 10; Tet1^−/−^ Saline, n = 7; Tet1^−/−^ HDM, n = 10–11. Mean ± SEM for each group is shown. Data are normally distributed and unpaired student t-tests were applied with Bonferroni corrections for multiple testing. *p < 0.05, **p < 0.01. ns represents not significant.

Next, we assessed the impact of Tet1 genotype on genes involved in pulmonary HDM induced immune responses, including IL-13, a central mediator of allergic asthma^45^. The expression levels of *Il13* in lung tissues were significantly elevated in Tet1^−/−^ mice compared to Tet1^+/+^ mice treated by HDM (Fig. 3a). HDM-induced *Il4* expression was similar between genotypes (Fig. 3a), consistent with the observed lack of differences in HDM-specific IgE levels (Fig. 2g). Among other pro-Th2 cytokines, HDM-treated Tet1^−/−^ mice had significantly higher *Il33* expression, and no significant changes in *Tslp*. In addition, we observed a significant increase in *Il17a* expression, but not *Il17f*. Expression of *Il6* and *Il1b*, two cytokines involved in Th17 differentiation, were not changed. Since genes involved in epithelial repair are upstream of the activation of the NF-κB pathway and they promote the generation of pro-Th2 and Th17 cytokines^46,47^, we next examined the expression of *Egfr*, *Tgfa*, and *Tff2* and found that the expression of *Egfr* and *Tff2* were both significantly increased HDM-treated Tet1^+/−^ mice (Fig. 3a). Consistent with changes in mRNA levels, we observed increased IL13 protein levels in BALF and significantly increased IL33 protein levels in lung homogenates of HDM-exposed Tet1^−/−^ mice (Fig. 3b). IL5 protein levels in BALF remained unchanged. Collectively, these data suggest that loss of Tet1 promotes the expression of pro-Th2 and Th17 cytokines and genes involved in epithelial damage and repair in the lungs.

### Loss of Tet1 increases the expression of *Muc5ac*

**Figure 4:**
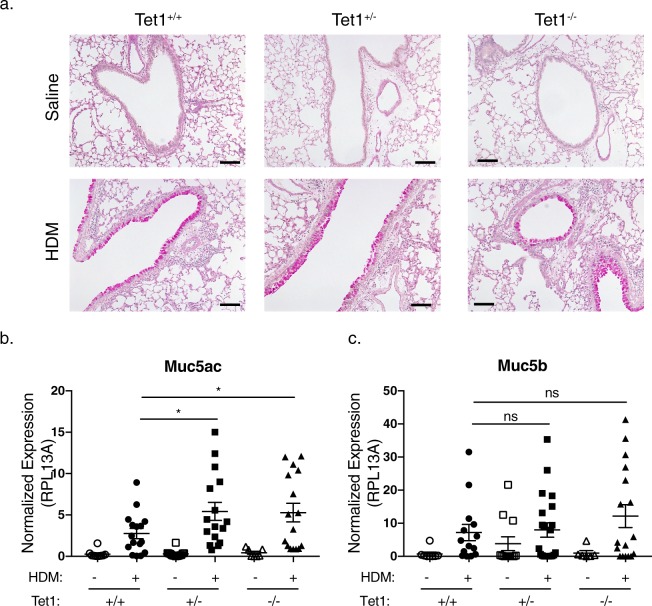
Tet1 deficiency increased the expression of *Muc5ac* in the lungs. (**a**) Periodic acid–Schiff (PAS) staining of lung sections. Representative images (4 slides per animal, 2–3 animals per group) are shown. (**b**) Expression of *Muc5ac* (**b**) and *Muc5b* (**c**) were measured by RT-qPCR. Expression values were normalized to Rpl13a. Mean ± SEM for each group is shown. Tet1^+/+^ Saline, n = 8–10; Tet1^+/+^ HDM, n = 14–16; Tet1^+/−^ Saline, n = 12–14; Tet1^+/−^ HDM, n = 16–21; Tet1^−/−^ Saline, n = 6–7; Tet1^−/−^ HDM, n = 15–17. Unpaired student t-tests (*Muc5ac*) or Mann Whitney tests (*Muc5b*) were applied. *p < 0.05. ns represents not significant. Bar represents 100 μm.

The strong Th2 response observed after HDM exposure induced significant mucus production; however, no significant differences in PAS stained large airways was observed between HDM challenged Tet1^+/+^_,_ Tet1^+/−^ and Tet1^−/−^ mice (representative images shown in Fig. 4a). To determine if Tet1 deficiency was associated with minor alterations in mucin composition, we assessed lung expression of *Muc5ac*, which is involved in allergen-induced AHR^48^ and increased in asthmatics^49,50^, as well as *Muc5b*, whose expression is decreased in asthmatics^49,50^ and functions to maintain normal mucus clearance^51^. The expression of *Muc5ac* was significantly increased in HDM-challenged Tet1^+/−^ mice and Tet1^−/−^ mice compared to Tet1^+/+^ mice (Fig. 4b), consistent with IL-13 BALF levels (Fig. 3b). No significant difference was observed for *Muc5b* expression (Fig. 4c). Taken together with increased lung IL-13 levels in HDM-treated Tet1^−/−^ mice, these data suggest that IL-13 mediated *Muc5ac* expression may contribute to the increased AHR in the HDM-treated Tet1^−/−^ mice.

### Lung transcriptomic analysis reveals interferon signaling as a major network upregulated in Tet1^−/−^ mice challenged by HDM

**Figure 5:**
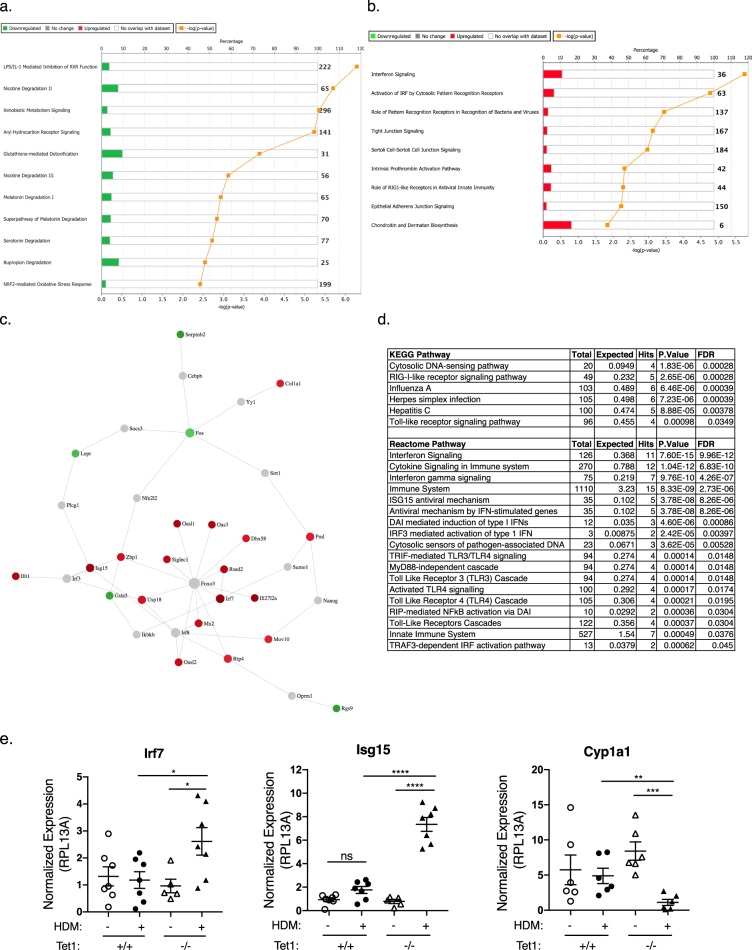
Pathway and protein network analysis of genes differentially expressed between HDM-challenged Tet1^+/+^ mice and Tet1^−/−^ mice. (**a**) Top 11 significantly enriched canonical pathways among the genes that were downregulated in HDM-treated Tet1^−/−^ mice. (**b**) Significantly enriched canonical pathways among the genes that were upregulated in HDM-treated Tet1^−/−^ mice. (**c**) Minimal network containing only direct protein-protein interactions between seed proteins from the input dataset. 133 genes differentially expressed genes were used as input, and the links between them indicate known protein-protein interactions. Green color marks genes with downregulated expression, red color marks genes with upregulated expression, grey color marks genes that are not included in the input dataset. (**d**) Significantly enriched pathways among genes included in c. (**e**) RT-qPCR validation of selected differentially expressed genes from RNA-seq. Tet1^+/+^ Saline, n = 6–7; Tet1^+/+^ HDM, n = 6–7; Tet1^−/−^ Saline, n = 5–6; Tet1^−/−^ HDM, n = 7. Expression levels were measured by RT-qPCR and were normalized to the expression of mRpl13a. Mean ± SEM for each group is shown. Data are normally distributed and unpaired student t-tests were applied with Bonferroni corrections for multiple testing. *p < 0.05, **p < 0.01, ***p < 0.001, ****p < 0.0001. ns represents not significant.

To gain mechanistic insight into the role of Tet1 in allergic airway responses, we performed an exploratory RNA-seq experiments on total lung RNA samples from saline- and HDM-challenged Tet1^+/+^ and Tet1^−/−^ mice (two animals in each group). Compared to Tet1^+/+^ mice treated with saline, saline-challenged Tet1^−/−^ mice showed differential expression in 104 genes, among which 22 genes were downregulated and 82 genes were upregulated (Supplementary Table S1). Pathways affected by the genes with elevated expression include Calcium signaling, Agranulocyte Adhesion and Diapedesis, Actin Cytoskeleton Signaling, TR/RXR Activation and Role of IL-17A in Psoriasis (Supplementary Table S2). In the HDM-treated mice, 72 genes showed reduced expression and 61 genes showed increased expression in Tet1^−/−^ compared to Tet1^+/+^ mice (Supplementary Table S3). Nineteen genes out of 133 genes overlap with genes dysregulated in the saline-treated mice, suggesting that these 19 genes are responsive to Tet1 but may not be specific to Tet1 deficiency in the context of HDM challenge. As our focus is to understand the impact of Tet1 on allergen-induced genes, these 19 genes were excluded from further pathway and gene network analysis. Among the downregulated genes in HDM-treated Tet1^−/−^ mice, the most significantly enriched pathways include LPS/IL-1 Mediated Inhibition of RXR Function, Nicotine Degradation II, Aryl Hydrocarbon Receptor Signaling, Xenobiotic Metabolism Signaling and Glutathione-mediated Detoxification (Fig. 5a and Supplementary Table S4A), all of which share genes such as *Cyp1a1*, *Gsta3*/*Gstm3*/*Gsto1*, *Fmo2*/*Fmo3* and *Aldh1a1*. Among the upregulated genes in HDM-treated Tet1^−/−^ mice, top significantly enriched pathways include Activation of IRF by Cytosolic Pattern Recognition Receptors, Interferon Signaling, Role of Pattern Recognition Receptors in Recognition of Bacteria and Viruses and Role of RIG1-like Receptors in Antiviral Innate Immunity (Fig. 5b and Supplementary Table S4A), all of which include components of the interferon (IFN) signaling pathway such as upstream regulator *Irf7* and downstream interferon-stimulated genes (ISGs) such as *Isg15*, *Ifit3* and *Oas1*/*Oas2/Oas3*. This was further supported by the upstream regulator analysis, where many components of IFN signaling, including type I/II/III interferon signaling, were identified as activated (Supplementary Table S4B). Accordingly, functions related to infectious diseases (viral infection and replication) were predicted to be repressed whereas functions related to inflammatory responses including T cell, B cell, macrophage and inflammatory diseases (rheumatic disease and multiple sclerosis) were predicted to be activated (Table 1 and Supplementary Table S4C).

**Table 1:** IPA predicted direction of change in functions by genes differentially expressed comparing HDM-challenged Tet1^−/−^ mice with Tet1^+/+^ mice.

Diseases or Functions Annotation	p-Value	Predicted Activation State*	Activation z-score
viral Infection	0.00000802	Decreased	−3.161
replication of virus	0.000284	Decreased	−3.012
replication of RNA virus	0.000353	Decreased	−2.738
malignant solid tumor	0.000245	Decreased	−2.564
replication of viral replicon	0.00000192	Decreased	−2.425
apoptosis of leukemia cell lines	0.00529	Decreased	−2.395
non-melanoma solid tumor	0.000669	Decreased	−2.319
replication of Herpesviridae	0.0000715	Decreased	−2.228
formation of reactive oxygen species	0.00897		−1.982
formation of osteoclasts	0.0107		−1.982
carcinoma	0.000888		−1.98
replication of vesicular stomatitis virus	0.000752		−1.974
replication of Flaviviridae	0.00193		−1.787
replication of Influenza A virus	0.0115		−1.551
quantity of lymphocytes	0.00936		−1.309
necrosis	0.00473		−0.915
cell death	0.00501		−0.731
quantity of mononuclear leukocytes	0.00502		−0.72
metabolism of terpenoid	0.00151		−0.711
apoptosis	0.00634		−0.711
infection of mammalia	0.000113		−0.523
liver lesion	0.00000152		−0.243
steroid metabolism	0.0105		−0.13
weight loss	0.00899		0
inflammation of respiratory system component	0.00745		0.068
remodeling of bone	0.00228		0.152
differentiation of leukemia cell lines	0.00492		0.152
quantity of cells	0.00885		0.2
phagocytosis by macrophages	0.00215		0.283
quantity of leukocytes	0.00575		0.293
binding of tumor cell lines	0.000332		0.357
T cell response	0.00492		0.401
activation of cells	0.00808		0.469
immune response of macrophages	0.00106		0.651
quantity of blood cells	0.00761		0.659
cell movement of tumor cell lines	0.00867		0.676
response of mononuclear leukocytes	0.00623		0.697
quantity of bone cells	0.00534		0.747
quantity of monocytes	0.00609		0.762
differentiation of tumor cell lines	0.00737		0.762
activation of leukocytes	0.0101		0.852
immune response of leukocytes	0.00288		0.87
production of antibody	0.00566		0.896
inflammatory response	0.00215		0.911
migration of tumor cell lines	0.00826		0.92
quantity of cytokine	0.00724		0.958
response of myeloid cells	0.00167		0.97
cell movement of fibroblasts	0.00977		1.067
proliferation of liver cells	0.00994		1.067
Rheumatic Disease	0.00123		1.119
apoptosis of squamous cell carcinoma cell lines	0.0013		1.154
quantity of connective tissue cells	0.00724		1.159
adhesion of tumor cell lines	0.00217		1.431
phagocytosis of cells	0.00654		1.432
cell movement	0.000223		1.503
phagocytosis	0.00333		1.741
quantity of phagocytes	0.0041		1.783
migration of cells	0.00129		1.898
immune response of cells	0.00708		1.931
ossification of bone	0.00304		1.982
cell death of macrophages	0.0116	Increased	2
relapsing-remitting multiple sclerosis	0.0000351	Increased	2.219
Multiple Sclerosis	0.00182	Increased	2.219

*Note: Functions with increased activation state are defined as those with z-score ≥2, and functions with decreased activation are defined as those with z-score ≤ −2.

We further performed network analysis to identify significantly enriched protein-protein interactions among the genes differentially expressed between Tet1^+/+^ and Tet1^−/−^ mice challenged by HDM. Among proteins encoded by differentially expressed genes (DEGs) in our dataset (seed nodes) and proteins directly interacting with them, there are several hub nodes centered around Irf7, Foxo3, Lepr, Pml, Cola1, and Fos. After trimming the network to only keep nodes that are necessary to connect the seed nodes (input genes), a smaller dense network including Irf7 and multiple ISGs (such as *Isg15*, *Mx2*, *Ifit1*, *Oas2*/*3*) was identified (Fig. 5c). Functional analyses (KEGG, Reactome and Molecular function) revealed that this sub-network is enriched for genes in Toll-like receptor signaling and IFN signaling (Fig. 5d and Supplementary Table S5). The expression differences in *Irf7*, *Isg15* and *Cyp1a1* were further validated by RT-qPCR (Fig. 5e). Collectively, these analyses identify IFN signaling as a major network that is significantly upregulated by loss of Tet1 following HDM challenges.

### TET1 regulates IFN and AhR signaling pathways in bronchial epithelial cells

**Figure 6:**
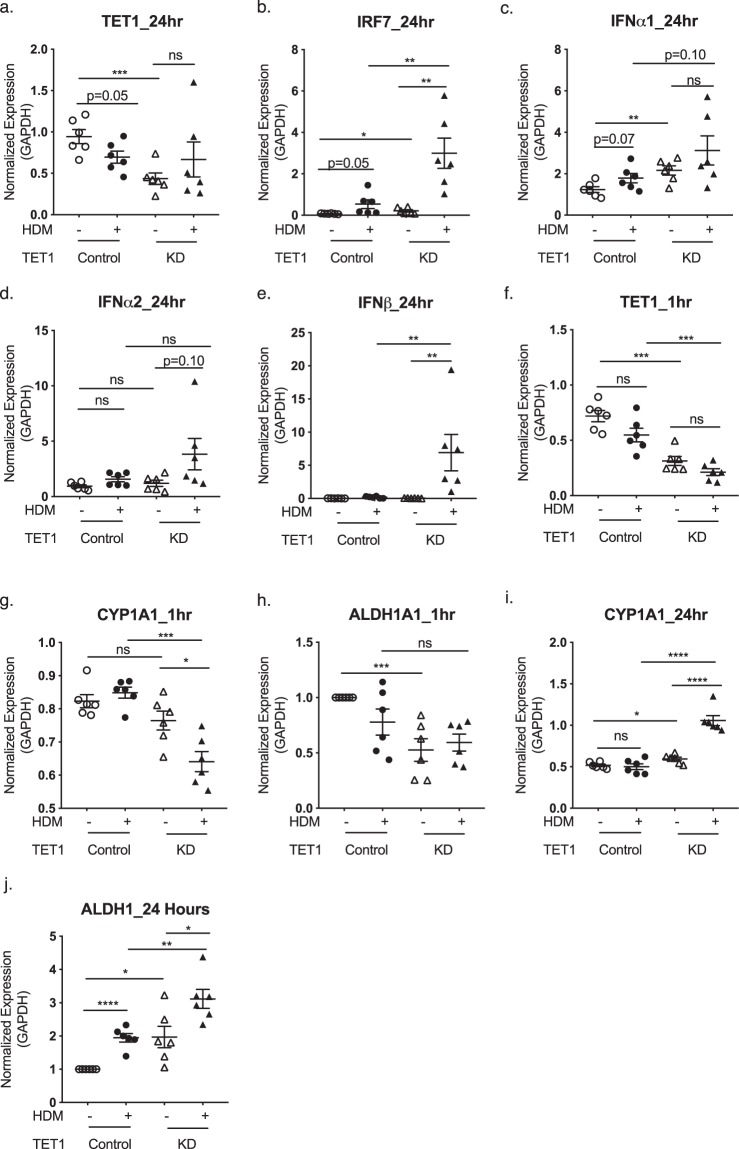
Knockdown of TET1 in human bronchial epithelial cells regulates the expression of genes in IFN and AhR pathways. Expression levels of *TET1*, *IRF7*, *IFNα1*, *IFNα2*, *IFNβ*, *CYP1A1*, and *ALDH1A1* in HBECs with or without TET1 knockdown and treatment of HDM were measured at indicated time points. Expression values of six technical replicates were normalized to the expression of GADPH. Mean ± SEM for each group is shown. Data are normally distributed and student t-tests were applied with Bonferroni corrections for multiple testing. *p < 0.05, **p < 0.01, ***p < 0.001, ****p < 0.0001. ns represents not significant.

As an important player in asthma pathogenesis^18,52,53^, airway epithelial cells are known to activate interferon and AhR signaling pathways following HDM challenges^54–62^. Because our transcriptomic analysis of lung RNA identified *Irf7* as a major component of IFN signaling regulated by Tet1 in mice challenged by HDM, we next determined whether such regulation of *Irf7* by *Tet1* occurs in bronchial epithelial cells treated by HDM. As shown in Fig. 6a,b, partial knockdown of *TET1* (~60%) in human bronchial epithelial cells (HBECs)^63^ significantly increased *IRF7* expression compared to siRNA treated controls at base line and following 24 hr HDM challenge (25 ug/ml). Similar increases were observed in the expression of IFNα isoforms^54^ (Fig. 6c,d) and IFNβ (Fig. 6e). IFNλ1/2 expression was not detectable.

In addition, we also observed downregulation of genes involved in AhR signaling, including *Cyp1a1* and *Aldh1a1* in lungs from HDM-treated Tet1^−/−^ mice comparing to Tet1^+/+^ mice. Since the expression of *CYP1A1* and *ALDHA1* (Phase I enzymes) represents an early response following activation of aryl hydrocarbon receptor signaling^64,65^, we examined the regulation of these genes by *TET1* in HBECs at both 1 hr and 24 hr time points. Downregulation of *TET1* in HBECs significantly reduced the expression of *CYP1A1* in the presence of HDM at 1 hr (Fig. 6f,h). For *ALDHA1*, we observed significant reduction in expression at 1 hr without HDM exposure and a trend of decrease with HDM exposure (Fig. 6h). Interestingly, both genes showed a significant increase following 24 hr exposure to HDM when *TET1* was knocked down (Fig. 6i,j), underscoring the temporally dynamic changes of these genes in response to HDM. In summary, our data showed that TET1 transcriptionally regulates the IFN and AhR signaling pathways in human bronchial epithelial cells following HDM challenges, consistent with the findings from the mouse model.

Next, as we observed relatively small but significant changes induced by Tet1 knockdown in HBECs without HDM at 24 hr time point (Fig. 6b,c,i,j), we examined whether activation of TET1 regulates the IFN signaling and AhR signaling pathways in HBECs without HDM challenges. Using a dCas9-SAM system to epigenetically activate the endogenous locus of *TET1*, we were able to increase *TET1* expression by ~14 fold (Supplementary Fig. S1a). The activation of TET1 expression led to significant downregulation of *IRF7* (Supplementary Fig. S1b) and other genes in IFN signaling pathway (Supplementary Fig. S1c, Supplementary Table S6 and S7). Genes involved in the AhR pathway were also regulated, including *CYP1A1* (Supplementary Table S6 and S7). A protein-protein interaction network including IRF7 and ISGs was identified by network analysis (Supplementary Fig. S1d, highlighted by blue circles). These data provide further support for the transcriptional regulation of IFN and AhR signaling pathways by TET1 in bronchial epithelial cells.

### Tet1 regulates IFN and AhR signaling in airway epithelial cells possibly through 5mC/5hmC, transcriptional factor binding and histone modification

**Figure 7:**
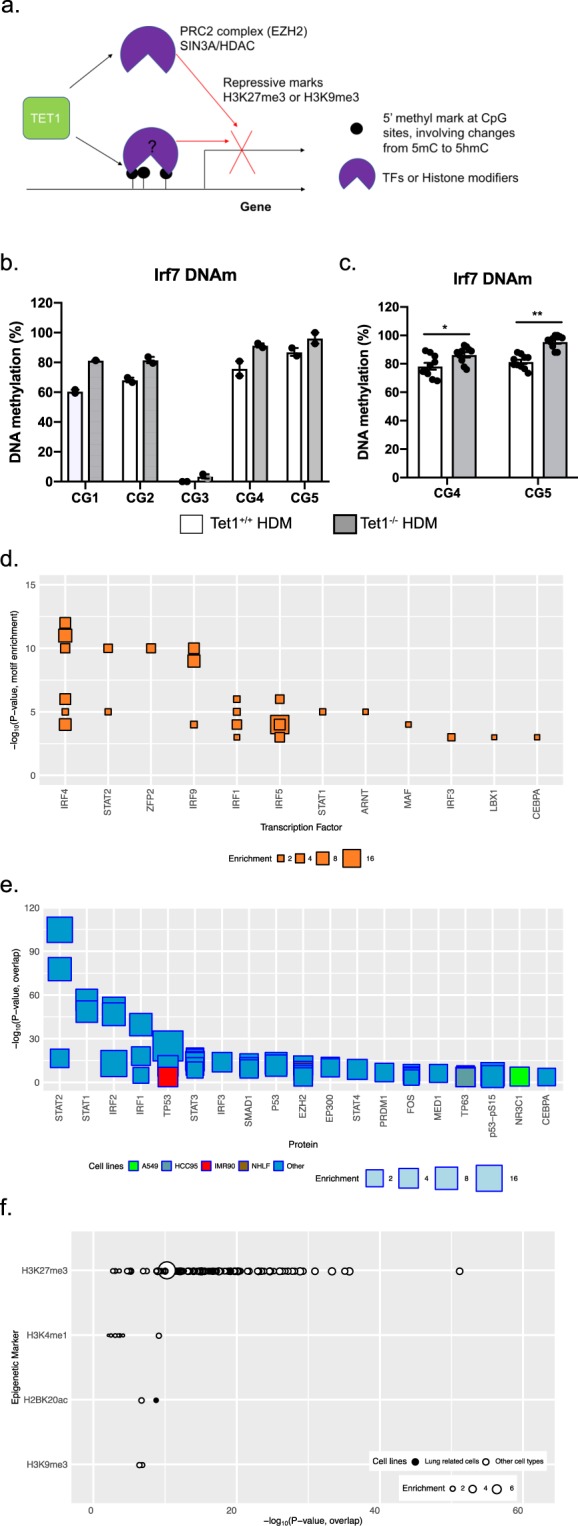
Possible mechanisms through which Tet1 regulates IFN and AhR signaling pathways. (**a**) Schematic showing two possible pathways that Tet1 utilizes to silence gene expression. (**b**) DNAm at 5 CpG sites across *Irf7* gene (mm10) from RRBS data at p < 0.05. Open bar: Tet1^+/+^ HDM; Grey bar: Tet1^−/−^ HDM. (**c**) Measurement of DNAm levels at CG4 and CG5 in b by bisulfite pyrosequencing. N = 10 for both groups and unpaired student t-tests were applied on normally distributed data. *p < 0.05, **p < 0.01. Open bar: Tet1^+/+^ HDM; Grey bar: Tet1^−/−^ HDM. (**d**) Transcription factor binding motif enrichment in promoter regions of Tet1-regulated genes in human bronchial epithelial cells. The top 30 significantly enriched binding motifs are grouped by their corresponding TFs (p < 10^−2^). The size of each square or circle indicates fold-enrichment over background. (**e**) Overlap significance between promoter regions (2 kb upstream transcription start site) of Tet1-regulated genes in bronchial epithelial cells and ChIP-seq datasets. The top 30 significant results are shown regardless of cell type, along with results from lung related datasets passing a p < 10^−6^ significant threshold. Lung-related cell types are shown in multi-color, and non-lung cell types are shown in dark blue. (**f**) Overlap significance between gene promoters and histone marks. The significance of the degree of overlap between promoters and each member of a large library of histone mark ChIP-seq datasets was estimated. Histone marks with at least one significant result (p < 10^−2^) are shown. The Y-axis indicates the histone mark, in decreasing order of significance. The X-axis indicates the significance (−log P-value) of the overlap of the given dataset. The size of each circle indicates the fold-enrichment relative to background.

Tet1 regulates gene expression by altering 5mC/5hmC at enhancers and promoters^21–24^, or binding to transcriptional co-factors such as Suz12 or the SIN3A complex to change histone modifications^31–34^ (Fig. 7a). We therefore performed genome-wide methylation analysis comparing lung DNA from HDM-treated Tet1^−/−^ and Tet1^+/+^ mice (two animals in each group). Our genome-wide analysis identified 10,087 differentially methylated CpG sites, which are located near 2,738 genes (Supplementary Table S8). The majority of these sites (10, 047 out of 10,087 CpG sites) showed increased DNAm in HDM-treated Tet1^−/−^ mice compared to Tet1^+/+^ mice, consistent with the established role of Tet1 in DNA demethylation. Although these pathways were not significant enriched (p > 0.05), we did find genes involved in Activation of IRF by Cytosolic Pattern Recognition Receptors/IFN signaling (*Tlr9*, *Irf8*, *Ifna4* and *Ifnar1*, etc) and LPS/IL-1 Mediated inhibition of RXR function and Aryl hydrocarbon receptor signaling (*Sod3*, *Nqo1*, *Gsto2*, *Aldhl1*/*Aldhl2*/*Aldha2*/*Aldh3a1*, etc) that were differentially methylated. However, we did not observe significant expression changes in these genes between HDM-treated Tet1^−/−^ and Tet1^+/+^ mice. In addition, *Irf7*, *Dhx58* and *Oas2* in IFN signaling pathway and *Aldh1a1*, *Fos*, *Gsto1* in AhR signaling pathway showed significant changes in DNAm at the level of p < 0.05. As shown in Fig. 7b, 5 CpG sites spanning the *Irf7* gene locus (chromosome coordinates in Supplementary Table S9) were found to be differentially methylated in RRBS and two of them were validated in additional samples (Fig. 7c). Collectively, our analysis support that the Tet1 regulates genes in IFN and AhR signaling pathways through DNAm, even though at certain loci such regulation may not correlate with detectable gene expression changes in mouse lungs.

We next explored other potential mechanisms that may account for the regulation of gene expression by TET1. To this end, we applied two complementary approaches to identify regulatory molecules present in the promoters of genes with expression levels dependent on TET1. First, we performed TF binding motif enrichment analysis using the HOMER software package^66^ and motifs taken from the Cis-BP database^67^, revealing an enrichment for interferon signaling (IRF and STAT) and AhR signaling (ARNT) TF binding motifs (Fig. 7d and Supplementary Table S10A). We next applied a previously described computational method^9^ to search for enriched ChIP-seq peaks for transcription factors, co-factors and histone marks at the promoter regions of genes transcriptionally regulated by TET1 in human bronchial epithelial cells. Consistent with our pathway, protein network and motif enrichment analyses, the top enriched TFs include significant players in IFN signaling (Fig. 7e and Supplementary Table S10B, STAT1/2/3/4 and IRF1/2/3/5/7/8/9). Many of these enriched motifs and ChIP-seq datasets are present in the promoter of *IRF7* (including IRF4/5, STAT1/3/4/5 and ARNT). Moreover, enriched binding was observed for EZH2 (Fig. 7e), a component of the PRC2 complex that generates H3K27me3 marks and is known to interact with TET1, was found. Consistent with the predicted binding of EZH2, H3K27me3 is by far the most significantly enriched histone marks around the promoter regions of TET1-regulated genes in HBECs (Fig. 7f and Supplementary Table S10C). For the *IRF7* and *CYP1A1* promoters, the binding of SIN3A, a known binding partner of TET1 to silence gene expression, was found (GSM803525). Histone modifications of H3K4, H3K9 and H3K27 that are indicative of promoter and enhancers were also found at the *IRF7* promoter in lung-related cells and tissues. Collectively, our functional genomics analyses suggest that TET1 may regulate genes in the IFN signaling pathway through interactions with TFs and histone modifiers in bronchial epithelial cells.

## Discussion

In this paper, we found that loss of Tet1 increased the severity of allergic airway disease, most notably AHR, in a mouse model exposed to the common allergen HDM. Accordingly, there was increased expression of *Muc5ac*, pro-Th2 and Th17 cytokine (e.g., *Il13*, *Il33* and *Il17a*), increased BALF eosinophilia, as well as increased genes involved in epithelial repair responses in the lungs (e.g. *Egfr* and *Tff2*). Integrative transcriptomic analysis, pathway and network analyses revealed the upregulation of genes in the IFN signaling pathway and downregulation of genes in the AhR signaling pathway in Tet1-deficient lungs challenged by HDM. A gene network centered around IRF7 was identified as the major hub among genes affected by Tet1. Consistent with data from the mouse lungs, knockdown of *TET1* in bronchial epithelial cells (HBECs), a major cell type that contributes to AHR and initiates lung inflammation, significantly upregulated expression of genes in the IFN signaling (*IRF7* and type I interferons) and altered genes in the AhR pathway (*CYP1A1* and *ALDHA1*) at base line and/or following HDM challenges. This transcriptional regulation of IFN and AhR pathways by Tet1 was further supported by transcriptomic analysis following activation of endogenous *TET1* expression in HBECs. To investigate possible mechanisms that Tet1 may regulate *Irf7* and other genes *in vivo*, we performed genome-wide methylation studies in mouse lung DNA and found changes in DNAm cross the *Irf7* gene and many other genes in IFN and AhR pathways. In addition, promoters of genes regulated by TET1 in HBECs are enriched for predicted binding of TFs that interact with TET1 or function in IFN and AhR signaling, as well as the presence of histone marks indicative of promoters and other regulatory elements. Collectively, our data suggest that TET1 prevents allergic airway inflammation through inhibition of the IFN signaling and activation of the AhR pathway in airway epithelial cells.

TET1 is known to regulate gene expression in many cell types including embryonic stem cells (ESC) and HEK293 cells^21–25^. This regulation could be achieved through 5hmC because TET1 can directly bind DNA to convert 5mC to 5hmC and 5hmC is known to promote DNA demethylation at enhancers and gene bodies by altering the local chromatin structure^68–75^. “Readers” of 5hmC marks activate downstream gene expression networks^21–24^. Consistent with this mechanism, we observed small but significant changes in DNAm within the *Irf7* gene in HDM-challenged-Tet1^−/−^ mice (Fig. 7b,c). Similar changes were observed for several genes in the IFN and AhR pathways that showed gene expression changes between HDM-treated Tet1^−/−^ and Tet1^+/+^ mice, although further validation studies are needed due to the limited number of samples included in RRBS. In addition, we also observed substantial DNAm changes (up to 60%) at other genes involved in IFN and AhR signaling whose expression changes were not detected following HDM challenges in the absence of Tet1. The lack of changes in gene expression may be due to the study of RNA expression in a mixture of lung cells instead of a single cell type, resulting in loss of signal. This could also explain why we did not observe expression changes in IFNα and IFNβ in total lung as in HBECs (differences in timing of measurement could be another reason). Alternatively, but not mutual exclusively, TET1 recruits histone modification enzymes (including OGT/SET1, SIN3A and EZH2 complexes) to regulate both active and repressive histone marks at promoters and enhancers in ESC and HEK293 cells and alter gene expression^31–34^. In support of this mechanism, we observed enriched binding of SIN3A and EZH2 within the promoters of Tet1-regulated genes in human bronchial epithelial cells and histone marks associated with active regulatory regions. Further studies investigating the spatial and temporal recruitment of TFs, histone marks, chromatin accessibility and 5mC/5hmC marks at genes regulated by Tet1 in airway epithelial cells, in combination with gene expression at the single-cell level, will significantly expand our understanding of Tet1’s gene regulatory roles in airway-related diseases.

In addition to Tet1, Tet2 and Tet3 may also contribute to the regulation of target genes due to their overlapping modulation of 5mC and 5hmC in other cell lines^76^. In addition, the DNA methyltransferases (DNMT1, 3A and 3B) are also enzymes that are important for maintenance and generation of 5mC marks. We found that *Tet1* and *Tet2* were significantly downregulated following HDM challenges in total lung RNA (a trend of downregulation was observed for Tet3), while *Dnmt1*, *Dnmt3a* and *Dnmt3b* remained unchanged (Supplementary Fig. S2). Therefore, the transcriptional responses to HDM in wildtype mouse lungs is likely mediated by TET, as opposed to DNMT proteins. This is different from a previous report using a relatively chronic HDM induced mouse model (1 i.p. sensitization by 100 ug HDM followed by 15 i.t. administration of 100 ug HDM over 5 weeks), where *Tet1* was significantly upregulated and *Dnmt3a* was significantly downregulated in HDM-treated mouse lungs^16^. This discrepancy is probably due to the differences between mouse models and the dynamic changes in these enzymes involved in DNAm in response to HDM. Importantly, loss of Tet1 did not significantly influence the expression of Tet2 and Tet3 in mouse lungs at base line or following HDM challenges (p = 0.21 and 0.35), suggesting that the exacerbated allergic airway inflammation we observed in HDM-challenged Tet1^−/−^ mice compared to Tet1^+/+^ mice were not mediated by Tet2 or Tet3. Interestingly, in the absence of Tet1, HDM treatment significantly reduced the expression of *Dnmt1*, *Dnmt3a* and *Dnmt3b* in addition to *Tet2* and *Tet3*, suggesting complex interactions between Tet proteins and DNMTs in mediating the responses to HDM. Although loss of *Tet1* significantly increased the expression of *Dnmt1* and *Dnmt3b* in saline-treated mice, *Dnmt1* and *Dnmt3b* were downregulated following HDM challenges (comparing HDM-challenged Tet1^−/−^ mice with Tet1^+/+^ mice). Because inhibition of DNMT activity using 5-aza before sensitization in a mouse model of experimental asthma alleviated allergic asthmatic features including lung inflammation, mucus production and AHR^77^, the exacerbated asthma features we observed in HDM-challenged Tet1^−/−^ mice compared to the HDM-challenged Tet1^+/+^ mice are unlikely due to the downregulation of *Dnmt1*, *Dnmt3a*, and *Dnmt3b*.

In the HDM-challenged mouse model, we observed an increase in the Th2 response in the absence of Tet1, while the IFN signaling pathway negatively regulated by Tet1 is mostly involved in viral responses and has been linked to asthma severity and exacerbation. As a master regulator of type I interferon-dependent immune responses^78^, IRF7 mediates airway epithelial responses to respiratory viral infection and was identified as a major hub gene connecting the IFN responses with virus-induced asthma exacerbations *in vivo*^79,80^. In addition, loss of IRF7 did not significantly alter HDM-induced allergic immune responses in mice^81^. However, it has been well established that respiratory viral infection leads to asthma exacerbation in human studies and in mouse models^82^. This is possibly due to the release of double-stranded DNA (dsDNA) triggered by viral infection that comes from NETosis (the formation of Neutrophil extracellular traps)^83^ in addition to HDM-induced dsDNA release due to tissue damage^84^. This free dsDNA binds to the TLR receptor and triggers signaling through IRF7 to promote downstream IFN responses^55^. We showed that loss of Tet1 transcriptionally mimics viral infection responses in mouse lungs and in human bronchial epithelial cells, which may promote the release of dsDNA and therefore contribute to an exacerbated IFN response. Therefore, we measured extracellular free dsDNA in BALF from our mouse models. Consistent with previous literature, there was significantly more extracellular dsDNA in BALF of mice challenged by HDM (Supplementary Fig. S3). Loss of Tet1 did not significantly change the amount of dsDNA in BALF. These data suggest that the enhanced asthma-related features in HDM-treated Tet1^−/−^ mice are not due to increased dsDNA ligand binding to toll receptors triggering downstream IFN signaling, but rather due to the loss of transcriptional modulation of components of the IFN signaling pathway by Tet1.

IFN signaling, including type I and III IFNs that can be produced by airway epithelial cells^54,55^, is known to regulate innate and adaptive type I and II immune responses (dendritic cells/Th1/Th2/Treg) following viral/bacterial/allergen challenges^85–88^ and contribute to asthma development/exacerbation^89,90^. However, we observed no obvious effects of Tet1 deletion on T effector/memory cell differentiation (data not shown), suggesting that the enhanced Th2 responses in HDM-challenged Tet1^−/−^ mice may not be due to the influences of IFN signaling on antigen presentation and T cell differentiation. In support of this, a very recent transcriptomic analysis of nasal epithelial brushing samples identified prominent activation of ISGs in adult asthmatics that is independent of viral infection, unrelated to type II inflammation, and associated with reduced lung function^91^. This is consistent with the presence of a T1-high group among severe asthmatics based on transcriptomic analysis^92^. Interestingly, *Irf7*^−/−^ and *Ifnar2*^−/−^ (deficient for type I IFN receptor) mice displayed normal dendritic cell development and allergic airway sensitization in response to HDM^81^, suggesting independence between type I IFN signaling and HDM-induced airway allergy when Tet1 is present. On the other hand, the AhR pathway, the second most significantly enriched pathway in our analysis, regulates airway epithelial multiciliogenesis^93^ and oxidative stress responses^60^, and contributes to asthma development and exacerbation^46,62,94^. Specifically, AhR ligands attenuate allergic airway inflammation in OVA-challenged animal models^94,95^, suggesting that down-regulation of the AhR pathway by Tet1 would increase the severity of HDM-induced allergic airway inflammation. Additionally, interactions between the IFN and AhR signaling pathways exist: increased IFN signaling by respiratory syncytial virus infection in airway epithelial cells induces protein degradation of transcription factor NRF2, which downregulates Nrf2-mediated expression of ARE-containing genes catalase and SOD1^96^. As the *CYP*, *ALDH* and *GST* genes identified in our RNA-seq analysis are also targets of Nrf2, the downregulation of the AhR pathway might be the consequence of upregulated IFN signaling in Tet1^−/−^ mice treated by HDM. Due to limited animal numbers included in the RNA-seq analysis, future studies will be performed to validate additional candidate genes in these pathways other than *Irf7*, *Isg15* and *Cyp1a1* (Fig. 5e). Whether the upregulation of IFN signaling (including upstream PRR signaling) and/or downregulation of AhR signaling pathways by Tet1 in airway epithelial cells directly leads to exacerbated HDM-induced allergic airway inflammation, and identification of the associated mechanisms, require further studies. Moreover, whether Tet1-mediated gene regulation would explain the interplay between viral infection and HDM-induced responses in airway epithelial cells and mouse lungs^59,97^ will be investigated in future studies.

In conclusion, we identified a novel role for Tet1 in asthma development. Our data suggest that the regulation of the IFN and AhR signaling pathways by Tet1 may contributes to the lung and airway epithelial responses to HDM challenges and the establishment of allergic airway inflammation. Similar to HDM, Tet1 expression is also responsive to other environmental exposures that contribute to asthma development and exacerbation, including diesel exhaust particles^8,10^ and cigarette smoke^98^. Therefore, our studies suggest that these exposures may contribute to asthma through the function of Tet1 on gene regulation. As TET1 can be modulated by small molecules (vitamin C, L-cysteine, 2-HG, etc) and miRNAs^34^, our findings may promote the development of new asthma therapies.

## Methods

### Murine model of allergic airway inflammation

Mice heterozygous for *Tet1*^*tm1.1Jae*^/J (B6/129S4) were purchased from Jackson Labs (Bar Harbor, ME) and littermates of wild type (Tet1^+/+^), heterozygous (Tet1^+/−^) and homozygous (Tet1^−/−^) for *Tet1*^*tm1.1Jae*^/J aged 8–12 weeks were utilized in the experiments. House dust mite (HDM) extract (*Dermatophagoides pteronyssinus*) was purchased from Greer Laboratories (Lenoir, NC). For an acute model of allergic airway inflammation, on days 0 and 7, mice were sensitized intraperitoneally with 100 μg of HDM (representing 33 µg of protein; 11 µg of Der p1; 5 EU of endotoxin) in 100 μL of PBS plus alum or an equivalent amount of PBS alone. On days 14, 19 and 21, mice were challenged intratracheally with 100 μg of HDM in 50 μL of PBS or PBS alone. On day 23, airway hyperresponsiveness (AHR) was analyzed, bronchoalveolar lavage fluid (BALF) were collected, blood was collected for measurement of serum proteins, and lung tissues were harvested for histology, immunohistochemistry and DNA/RNA extraction. All mice were maintained in a barrier facility at Cincinnati Children’s Hospital Medical Center and handled under Institutional Animal Care and Use Committee-approved procedures (protocol approved by CCHMC IACUC committee). All methods were performed in accordance with the relevant guidelines and regulations.

### Measurement of airway hyperresponsiveness (AHR)

Invasive measurements of AHR were made on a flexiVent apparatus (Scireq, Montreal, Canada). Mice were anesthetized with Ketamaine, Xylazine, and Acepromazine (100, 20 and 10 mg/ml mixed at a ratio of 4:1:1). Mouse tracheas were cannulated with a 20-gauge blunt needle and the mice were ventilated at 150 breaths/min, 3.0 cm water positive end expiratory pressure. Two total lung capacity perturbations were then performed for airway recruitment before baseline measurement and subsequent methacholine challenges were performed. Dynamic resistance (R) and compliance (C) were determined by fitting the data to a single compartment model of airway mechanics where Ptr = RV + EV + Po, and Ptr = tracheal pressure, V = volume, E = elastance, Po is a constant and C = 1/E. Measurements were made using a 1.25 s, 2.5 Hz volume-driven oscillation applied to the airways by a computer-controlled piston (SnapShot perturbation). PBS or methacholine was aerosolized for 15 s (Aeroneb ultrasonic nebulizer) followed by 15 s of ventilation and a SnapShot perturbation was performed followed by 10 s of ventilation. Twelve SnapShot/ventilation cycle measurements were made. The procedure was repeated for 0, 6.25, 12.5, 25, 50, and 100 mg/ml concentrations of methacholine. The maximum R value and minimum C value with a coefficient of determination of 0.9 or greater (as determined by the flexiVent software) were used to determine the dose-response curve.

### Bronchoalveolar lavage fluid (BALF) collection and analysis

Bronchoalveolar lavage was performed by cannulation of the trachea. The lungs were lavaged with 1 ml PBS +0.5% BSA +2 mM EDTA. The collected BALF was centrifuged and the total cell numbers counted with a hemacytometer. Cells were spun onto slides and stained with the HEMA3 stain kit (Fisher Scientific, Kalamazoo, MI). 400 cells were counted and the total number and % of each cell type were calculated.

### Histology and immunohistochemistry

The left lobe of the lung was fixed in formalin, paraffin embedded and cut into 5 µm sections. Sections were stained with hematoxylin and eosin (H&E) or PAS according to the manufacturer’s recommendations (PolyScientific).

### Lung homogenate

Frozen lung tissue was homogenised mechanically (OmniPrep Rotor Stator Generator, Omni International, USA) and enzymatically by addition of RIPA lysis buffer (MilliporeSigma, Burlington, MA) with protease inhibitors (MilliporeSigma) added. Samples were centrifuged and supernatants were saved. Total protein concentration was determined using BCA assay (ThermoFisher Scientific).

### ELISA in serum, BALF and total lung homogenate

Total serum IgE and IgG1, HDM-specific IgE and IgG1 levels were measured as previously described^99,100^. For measurement of HDM-specific IgE and IgG1 levels, wells were coated with 0.01% HDM (Greer Laboratories) overnight. Serum was diluted 1:5 for IgE and 1:2000 for IgG1. After 2 hours of incubation, plates were washed, and either horseradish peroxidase–conjugated anti-mouse IgG1 (X56; 1:1000; BD Biosciences PharMingen, San Jose, Calif) or biotin–anti-mouse IgE (R35-118; 1:250; PharMingen) was added for 1 hour, followed by an incubation with streptavidin–horseradish peroxidase (R&D DY998; 1:200) in the case of IgE. BALF cytokine were also quantified as previously described^99,100^. IL13 protein levels were measured using Mouse IL-13 DuoSet ELISA kit (R&D Systems, 62.5–4,000 pg/mL) per the manufacturer’s instructions. IL5 was measured using Mouse IL-5 ELISA MAX™ Standard kit (Biolegend, 7.8 pg/mL to 500 pg/mL) per the manufacturer’s instructions. IL33 protein levels in lung homogenates were measured using Mouse IL-33 DuoSet ELISA kit (R&D Systems, 15.6 pg/mL–1000 pg/mL) per the manufacturer’s instructions. The results were then presented in relation to total protein concentration for each sample.

### Activation and knockdown of TET1 expression in human bronchial epithelial cells

Human bronchial epithelial cells (HBECs) is a cell line obtained from Dr. John Minna’s lab at UT Southwestern Medical Center^63^. This cell line was created from bronchial specimens from areas of the lung histologically not involved with lung cancer by ectopic expression of *Cdk4* and *hTERT*. It is not carcinogenic, has epithelial morphology and the expression of epithelial markers, and has an intact p53 checkpoint pathway. Previous cytogenetic analysis and array comparative genomic hybridization profiling identified duplication of parts of chromosomes 5 and 20. Microarray gene expression analysis demonstrated that this cell line clustered with non-immortalized bronchial cells, distinct from lung cancer cell lines.

HBECs were grown to 70–90% confluence in keratinocyte serum free medium supplemented with human recombinant Epidermal Growth Factor and Bovine Pituitary Extract (Life Technologies, Carlsbad, CA). For the CRISPR plasmid Activation of TET1, control plasmid and TET1 CRISPR activation plasmid (Santa Cruz Biotechnology, Dallas, TX) was transfected into HBECs using Lipofectamine® 2000 (Thermo Fisher Scientific, Florence, KY) and Opti-MEM medium according to manufacturer’s protocol. Briefly, 1 ug plasmid DNA was diluted with Opti-MEM. Lipofectamine® 2000 Reagent was diluted, in a separate tube, with Opti-MEM. The DNA plasmid dilution was then mixed with the Lipofectamine® 2000 dilution (1:1 ratio) for 5 minutes. The DNA-lipid complex was added to cells in a 12-well plate and incubated for 48 hours before harvest. For the siRNA knockdown of TET1, two siRNA targeting TET1 products were combined to increase knockdown efficiency (Thermo Fisher scientific and Santa Cruz). Lipofectamine® RNAiMAX™ Transfection Reagent (Thermo Fisher Scientific, Florence, KY) was used.

### RNA extraction and RT-qPCR

Mouse lungs were homogenized using ceramic beads (Omni Inc, Kennesaw, Georgia) and the Omni Bead Ruptor 24 (Omni Inc, Kennesaw Georgia). Total RNA was isolated from homogenized mouse lung using Trizol (Invitrogen, Carlsbad, CA) and RNA was purified using the RNeasy Mini Kit (Qiagen, Valencia, CA) according to manufacturer’s instructions. DNase treatment was performed (Qiagen, Valencia, CA) before being reverse transcribed with SuperScript™ IV Vilo Master Mix™ (Invitrogen, Carlsbad, CA). Reverse-Transcriptase quantitative PCR (RT-qPCR) analysis was done using LightCycler FastStart DNA master SYBR green I as a ready-to-use reaction mixture (Roche, Indianapolis, Indiana). Targeted genes were amplified from cDNA using the primers listed in Supplementary Table S11 and gene expression was normalized to Rpl13a (mouse) or GAPDH (human). Melting curves have been generated for all assays and one product for each gene has been identified.

### RNA-seq library preparation and sequencing

1 ug RNA per sample was used for each RNA-seq experiment. Samples from two animals in each group (Tet1^+/+^ Saline, Tet1^+/+^ HDM, Tet1^−/−^ Saline, Tet1^−/−^ HDM) were included. Two technical replicates from the Tet1 activation experiment in HBECs were analyzed. The purity and concentration of the isolated RNA was quantified using the Nanodrop 1000 (Thermo Fisher Scientific, Wilmington, DE) and 2100 Bioanalyzer (Agilent technologies, Santa Clara, CA). A total of 1 μg RNA for each sample with RNA integrity number (RIN) values greater than 8 was used for library construction. Briefly, mRNA was purified from total RNA using poly-T oligo-attached magnetic beads. Following purification, the mRNA was fragmented and reverse-transcribed to create the final cDNA library in accordance with the protocol for the mRNA-Seq sample preparation kit (Illumina, San Diego, USA). cDNA libraries were sequenced on an Illumina HiSeq™ 2000 platform, and reads were generated in 100-bp single-end format.

### RNA-seq data analysis and functional genomics analyses

All fastq files were adapter trimmed prior to alignment. Files were aligned against Human (GRch37/Ensembl) or Mouse reference genome (GRCm38/mm10). Sequence alignment was performed using Bowtie2 and RSEM^101^. Post-alignment, raw read counts were extracted and normalized to account for difference in read depth using DESeq^102^. Following normalization, gene read counts were filtered to remove low read values: maximum Reads Per Kilobase of transcript, per Million mapped reads (RPKM) across all samples must be ≥1. Differentially expressed genes (DEGs) were identified using DESeq, and genes with an FDR adjusted p-value (q-value) <0.01 and fold of change ≥1.2, were considered significant. P values were calculated by student t test for each individual gene, and Benjamini and Hochberg correction was applied to generate FDR (q-value).

Gene ontology and pathway analysis were performed in Ingenuity Pathway Analysis (IPA, Ingenuity Systems, Redwood City, CA) and a cutoff of 0.05 was used for statistical significance in IPA analysis. Protein-protein interaction networks and modules were exacted using a web-based tool^103,104^ and 1^st^ order subnetwork or minimal networks were shown. Enriched transcription factor (TF) interaction and histone marks at promoter regions (2 kb upstream TSS) of differentially expressed genes over a background data set (containing genes that are not differentially expressed from the same RNA-seq analysis) were identified as previously described^10^.

### Reduced representation bisulfite sequencing (RRBS) sample processing, alignment, methylation calling and differential methylation analysis

After mouse lungs were homogenized (see above in RNA extraction), DNA was extracted using DNeasy Blood and Tissue Kits (Qiagen, Valencia, CA) per the manufacturer’s instructions. Samples from two animals in Tet1^+/+^ HDM and Tet1^−/−^ HDM groups were included. For RRBS, 100 ng gDNA and 10 units *Msp*I restriction enzyme were added in 20 ul reaction, and then incubated at 37 °C for 2 hours. *Msp*I digests the CCGG sites and generated CG overhang at 5′ end, which were end-repaired and A-tailed using the T4 DNA polymerase and Klenow fragments, and subsequently ligated with Illumina adapters. The DNA samples were then treated with sodium bisulfite which converts unmethylated cytosine to uracil while methylated cytosine remains unaffected. Size selection was performed to obtain the optimal fragments for genome coverage and remove restriction fragments that failed to ligate with the adapter. Purified DNA then underwent the minimum number of cycles to produce an evenly represented library. 20 million SE75 reads were generated from Illumina HiSeq 2500.

FASTQ files were obtained from the DNA Sequencing and genotyping Core facility of CCHMC. Quality control steps were performed to determine overall quality of the reads from the FASTQ files. Upon passing basic quality matrices, the reads were trimmed to remove adapters and low-quality reads using Trim galore. The trimmed reads were then mapped to the bisulfite converted mouse reference genome mm10 using bismark, which created a summary of alignment, overall methylation profile and output BAM files. Next, the methylation levels of all the covered CpG sites were obtained using methylation extractor option of Bismark tool. Their association with a CpG-Island and/or a promoter and read coverage of these sites were calculated using R. An R package called MethylKit was used to identify differentially methylated sites^105^. P values were calculated by logistic regression for each individual CpG site. The sliding linear model (SLIM method^106^) was applied to generate FDR (q-value). The CpG sites were annotated to genes using a custom PERL script.

### Bisulfite Pyrosequencing

A total of 100 ng genomic DNA was subjected to sodium bisulfite treatment. Standard PCR amplification reactions were performed to amplify targeted gene fragments at an annealing temperature of 50 °C before being subjected to pyrosequencing. The generated pyrograms were automatically analyzed using PyroMark analysis software (Qiagen, Valencia, CA, USA). Pyrosequencing assay design and genomic coordinates are documented in Supplementary Table S9.

### Statistical analysis

All statistical analyses were performed using GraphPad Prism 7.0 with a p value cutoff of 0.05 considered significant. Normality of data were checked using Shapiro-Wilk normality test and α was set to be 0.05. When data are normally distributed, groups were compared using student’s t-tests. For data that are not normally distributed, Mann Whitney tests were performed. For multiple comparisons, Bonferroni post hoc corrections were used.

## Supplementary information

Supplementary Information

Table S1

Table S2

Table S3

Table S4

Table S5

Table S6

Table S7

Table S8

Table S10

## Data Availability

RNA-seq and RRBS data has be deposited in GEO (GSE124922).

## References

[1] National Survey of Children’s Health. NSCH 2011/12. Data query from the Child and Adolescent Health Measurement Initiative, Data Resource Center for Child and Adolescent Health website.

[2] Centers for Disease Control and Prevention. National Center for Health Statistics. National Health Interview Survey, 2015. Analysis by the American Lung Association Epidemiology and Statistics Unit using SPSS software.

[3] Barnett SB, Nurmagambetov TA (2011). Costs of asthma in the United States: 2002–2007. The Journal of allergy and clinical immunology.

[4] Rank MA (2012). Asthma expenditures in the United States comparing 2004 to 2006 and 1996 to 1998. Am J Manag Care.

[5] Nunes C, Pereira AM, Morais-Almeida M (2017). Asthma costs and social impact. Asthma Res Pract.

[6] Morrow T (2007). Implications of pharmacogenomics in the current and future treatment of asthma. Journal of managed care pharmacy: JMCP.

[7] Pelaia G, Vatrella A, Maselli R (2012). The potential of biologics for the treatment of asthma. Nature reviews. Drug discovery.

[8] Somineni HK (2016). Ten-eleven translocation 1 (TET1) methylation is associated with childhood asthma and traffic-related air pollution. The Journal of allergy and clinical immunology.

[9] Zhang X (2017). Nasal DNA methylation differentiates corticosteroid treatment response in pediatric asthma: A pilot study. PloS one.

[10] Zhang X (2018). Nasal DNA methylation is associated with childhood asthma. Epigenomics.

[11] Ji H (2016). Air pollution, epigenetics, and asthma. Allergy Asthma Clin Immunol.

[12] Oh S, Ji H, Barzman D, Lin PI, Hutton J (2015). Pediatric asthma and autism-genomic perspectives. Clin Transl Med.

[13] Xu CJ (2018). DNA methylation in childhood asthma: an epigenome-wide meta-analysis. Lancet Respir Med.

[14] Yu Q (2012). DNA methyltransferase 3a limits the expression of interleukin-13 in T helper 2 cells and allergic airway inflammation. Proceedings of the National Academy of Sciences of the United States of America.

[15] Brand S (2012). DNA methylation of TH1/TH2 cytokine genes affects sensitization and progress of experimental asthma. J Allergy Clin Immunol.

[16] Cheng RY (2014). Alterations of the lung methylome in allergic airway hyper-responsiveness. Environmental and molecular mutagenesis.

[17] Shang Y (2013). Epigenetic alterations by DNA methylation in house dust mite-induced airway hyperresponsiveness. Am J Respir Cell Mol Biol.

[18] Holgate ST (2011). The sentinel role of the airway epithelium in asthma pathogenesis. Immunol Rev.

[19] Lambrecht BN, Hammad H (2014). Allergens and the airway epithelium response: Gateway to allergic sensitization. The Journal of allergy and clinical immunology.

[20] Gras D, Chanez P, Vachier I, Petit A, Bourdin A (2013). Bronchial epithelium as a target for innovative treatments in asthma. Pharmacology & therapeutics.

[21] Yildirim O (2011). Mbd3/NURD complex regulates expression of 5-hydroxymethylcytosine marked genes in embryonic stem cells. Cell.

[22] Spruijt CG (2013). Dynamic readers for 5-(hydroxy)methylcytosine and its oxidized derivatives. Cell.

[23] Mellen M, Ayata P, Dewell S, Kriaucionis S, Heintz N (2012). MeCP2 binds to 5hmC enriched within active genes and accessible chromatin in the nervous system. Cell.

[24] Sayeed SK, Zhao J, Sathyanarayana BK, Golla JP, Vinson C (2015). C/EBPbeta (CEBPB) protein binding to the C/EBP|CRE DNA 8-mer TTGC|GTCA is inhibited by 5hmC and enhanced by 5mC, 5fC, and 5caC in the CG dinucleotide. Biochim Biophys Acta.

[25] Grosser C, Wagner N, Grothaus K, Horsthemke B (2015). Altering TET dioxygenase levels within physiological range affects DNA methylation dynamics of HEK293 cells. Epigenetics.

[26] Tahiliani M (2009). Conversion of 5-methylcytosine to 5-hydroxymethylcytosine in mammalian DNA by MLL partner TET1. Science.

[27] He YF (2011). Tet-mediated formation of 5-carboxylcytosine and its excision by TDG in mammalian DNA. Science.

[28] Ito S (2011). Tet proteins can convert 5-methylcytosine to 5-formylcytosine and 5-carboxylcytosine. Science.

[29] Pastor WA, Aravind L, Rao A (2013). TETonic shift: biological roles of TET proteins in DNA demethylation and transcription. Nature reviews. Molecular cell biology.

[30] Wu H, Zhang Y (2014). Reversing DNA methylation: mechanisms, genomics, and biological functions. Cell.

[31] Williams K (2011). TET1 and hydroxymethylcytosine in transcription and DNA methylation fidelity. Nature.

[32] Zhang Q (2015). Tet2 is required to resolve inflammation by recruiting Hdac2 to specifically repress IL-6. Nature.

[33] Cartron PF (2013). Identification of TET1 Partners That Control Its DNA-Demethylating Function. Genes Cancer.

[34] Delatte B, Deplus R, Fuks F (2014). Playing TETris with DNA modifications. Embo J.

[35] Vincent JJ (2013). Stage-specific roles for tet1 and tet2 in DNA demethylation in primordial germ cells. Cell Stem Cell.

[36] Costa Y (2013). NANOG-dependent function of TET1 and TET2 in establishment of pluripotency. Nature.

[37] Huang Y (2014). Distinct roles of the methylcytosine oxidases Tet1 and Tet2 in mouse embryonic stem cells. Proceedings of the National Academy of Sciences of the United States of America.

[38] Yamaguchi S, Shen L, Liu Y, Sendler D, Zhang Y (2013). Role of Tet1 in erasure of genomic imprinting. Nature.

[39] Kaas GA (2013). TET1 controls CNS 5-methylcytosine hydroxylation, active DNA demethylation, gene transcription, and memory formation. Neuron.

[40] Rudenko A (2013). Tet1 is critical for neuronal activity-regulated gene expression and memory extinction. Neuron.

[41] Zhang RR (2013). Tet1 regulates adult hippocampal neurogenesis and cognition. Cell Stem Cell.

[42] Huang H (2013). TET1 plays an essential oncogenic role in MLL-rearranged leukemia. Proceedings of the National Academy of Sciences of the United States of America.

[43] Tsagaratou A, Lio CJ, Yue X, Rao A (2017). TET Methylcytosine Oxidases in T Cell and B Cell Development and Function. Front Immunol.

[44] Marques-Garcia F, Marcos-Vadillo E (2016). General and Specific Mouse Models for Asthma Research. Methods Mol Biol.

[45] Wills-Karp M (1998). Interleukin-13: central mediator of allergic asthma. Science.

[46] Brandt EB, Myers JM, Ryan PH, Hershey GK (2015). Air pollution and allergic diseases. Curr Opin Pediatr.

[47] Wills-Karp M (2012). Trefoil factor 2 rapidly induces interleukin 33 to promote type 2 immunity during allergic asthma and hookworm infection. The Journal of experimental medicine.

[48] Evans CM (2015). The polymeric mucin Muc5ac is required for allergic airway hyperreactivity. Nat Commun.

[49] Fahy JV (2002). Goblet cell and mucin gene abnormalities in asthma. Chest.

[50] Woodruff PG (2009). T-helper type 2-driven inflammation defines major subphenotypes of asthma. American journal of respiratory and critical care medicine.

[51] Roy MG (2014). Muc5b is required for airway defence. Nature.

[52] Kuperman DA (2002). Direct effects of interleukin-13 on epithelial cells cause airway hyperreactivity and mucus overproduction in asthma. Nature medicine.

[53] Willis-Owen SA, Cookson WO, Moffatt MF (2009). Genome-wide association studies in the genetics of asthma. Curr Allergy Asthma Rep.

[54] Khaitov MR (2009). Respiratory virus induction of alpha-, beta- and lambda-interferons in bronchial epithelial cells and peripheral blood mononuclear cells. Allergy.

[55] Hermant P, Michiels T (2014). Interferon-lambda in the context of viral infections: production, response and therapeutic implications. J Innate Immun.

[56] de Weerd NA, Nguyen T (2012). The interferons and their receptors–distribution and regulation. Immunol Cell Biol.

[57] Ioannidis I, Ye F, McNally B, Willette M, Flano E (2013). Toll-like receptor expression and induction of type I and type III interferons in primary airway epithelial cells. J Virol.

[58] Sopel N, Pflaum A, Kolle J, Finotto S (2017). The Unresolved Role of Interferon-lambda in Asthma Bronchiale. Front Immunol.

[59] Golebski K (2014). High degree of overlap between responses to a virus and to the house dust mite allergen in airway epithelial cells. PloS one.

[60] Chiba T (2011). Arylhydrocarbon receptor (AhR) activation in airway epithelial cells induces MUC5AC via reactive oxygen species (ROS) production. Pulm Pharmacol Ther.

[61] Tsai MJ (2014). Aryl hydrocarbon receptor (AhR) agonists increase airway epithelial matrix metalloproteinase activity. J Mol Med (Berl).

[62] Chiba T, Chihara J, Furue M (2012). Role of the Arylhydrocarbon Receptor (AhR) in the Pathology of Asthma and COPD. J Allergy (Cairo).

[63] Ramirez RD (2004). Immortalization of human bronchial epithelial cells in the absence of viral oncoproteins. Cancer research.

[64] Dietrich C (2016). Antioxidant Functions of the Aryl Hydrocarbon Receptor. Stem Cells Int.

[65] Liska DJ (1998). The detoxification enzyme systems. Altern Med Rev.

[66] Heinz S (2010). Simple combinations of lineage-determining transcription factors prime cis-regulatory elements required for macrophage and B cell identities. Molecular cell.

[67] Weirauch MT (2014). Determination and inference of eukaryotic transcription factor sequence specificity. Cell.

[68] Stroud H, Feng S, Morey Kinney S, Pradhan S, Jacobsen SE (2011). 5-Hydroxymethylcytosine is associated with enhancers and gene bodies in human embryonic stem cells. Genome biology.

[69] Serandour AA (2012). Dynamic hydroxymethylation of deoxyribonucleic acid marks differentiation-associated enhancers. Nucleic acids research.

[70] Lister R (2013). Global epigenomic reconfiguration during mammalian brain development. Science.

[71] Lu F, Liu Y, Jiang L, Yamaguchi S, Zhang Y (2014). Role of Tet proteins in enhancer activity and telomere elongation. Genes Dev.

[72] Hon GC (2014). 5mC oxidation by Tet2 modulates enhancer activity and timing of transcriptome reprogramming during differentiation. Molecular cell.

[73] Bogdanovic O (2016). Active DNA demethylation at enhancers during the vertebrate phylotypic period. Nature genetics.

[74] Kranzhofer DK (2016). 5′-Hydroxymethylcytosine Precedes Loss of CpG Methylation in Enhancers and Genes Undergoing Activation in Cardiomyocyte Maturation. PloS one.

[75] Mahe EA (2017). Cytosine modifications modulate the chromatin architecture of transcriptional enhancers. Genome Res.

[76] Putiri EL (2014). Distinct and overlapping control of 5-methylcytosine and 5-hydroxymethylcytosine by the TET proteins in human cancer cells. Genome biology.

[77] Guajardo JR (2005). Altered gene expression profiles in nasal respiratory epithelium reflect stable versus acute childhood asthma. J Allergy Clin Immunol.

[78] Honda K (2005). IRF-7 is the master regulator of type-I interferon-dependent immune responses. Nature.

[79] Bosco A, Ehteshami S, Panyala S, Martinez FD (2012). Interferon regulatory factor 7 is a major hub connecting interferon-mediated responses in virus-induced asthma exacerbations *in vivo*. The Journal of allergy and clinical immunology.

[80] Bosco A, Wiehler S, Proud D (2016). Interferon regulatory factor 7 regulates airway epithelial cell responses to human rhinovirus infection. BMC Genomics.

[81] Marichal T (2010). Interferon response factor 3 is essential for house dust mite-induced airway allergy. The Journal of allergy and clinical immunology.

[82] Custovic A (2013). EAACI position statement on asthma exacerbations and severe asthma. Allergy.

[83] Toussaint M (2017). Host DNA released by NETosis promotes rhinovirus-induced type-2 allergic asthma exacerbation. Nature medicine.

[84] Chan TK (2016). House dust mite-induced asthma causes oxidative damage and DNA double-strand breaks in the lungs. The Journal of allergy and clinical immunology.

[85] Gonzales-van Horn SR, Farrar JD (2015). Interferon at the crossroads of allergy and viral infections. Journal of leukocyte biology.

[86] Lundie RJ (2016). A central role for hepatic conventional dendritic cells in supporting Th2 responses during helminth infection. Immunol Cell Biol.

[87] Duerr CU (2016). Type I interferon restricts type 2 immunopathology through the regulation of group 2 innate lymphoid cells. Nature immunology.

[88] Webb LM (2017). Type I interferon is required for T helper (Th) 2 induction by dendritic cells. Embo J.

[89] Koch S, Finotto S (2015). Role of Interferon-lambda in Allergic Asthma. J Innate Immun.

[90] Altman MC (2017). Interferon response to respiratory syncytial virus by bronchial epithelium from children with asthma is inversely correlated with pulmonary function. The Journal of allergy and clinical immunology.

[91] Bhakta NR (2018). IFN-stimulated Gene Expression, Type 2 Inflammation, and Endoplasmic Reticulum Stress in Asthma. American journal of respiratory and critical care medicine.

[92] Modena BD (2017). Gene Expression Correlated with Severe Asthma Characteristics Reveals Heterogeneous Mechanisms of Severe Disease. American journal of respiratory and critical care medicine.

[93] Villa M (2016). The aryl hydrocarbon receptor controls cyclin O to promote epithelial multiciliogenesis. Nat Commun.

[94] Beamer CA, Shepherd DM (2013). Role of the aryl hydrocarbon receptor (AhR) in lung inflammation. Semin Immunopathol.

[95] Xu T (2015). Aryl Hydrocarbon Receptor Protects Lungs from Cockroach Allergen-Induced Inflammation by Modulating Mesenchymal Stem Cells. Journal of immunology.

[96] Komaravelli N, Ansar M, Garofalo RP, Casola A (2017). Respiratory syncytial virus induces NRF2 degradation through a promyelocytic leukemia protein - ring finger protein 4 dependent pathway. Free Radic Biol Med.

[97] Akbarshahi H (2018). House dust mite impairs antiviral response in asthma exacerbation models through its effects on TLR3. Allergy.

[98] Coulter JB, O’Driscoll CM, Bressler JP (2013). Hydroquinone increases 5-hydroxymethylcytosine formation through ten eleven translocation 1 (TET1) 5-methylcytosine dioxygenase. The Journal of biological chemistry.

[99] Brandt EB (2015). Exposure to allergen and diesel exhaust particles potentiates secondary allergen-specific memory responses, promoting asthma susceptibility. The Journal of allergy and clinical immunology.

[100] Brandt EB (2013). Diesel exhaust particle induction of IL-17A contributes to severe asthma. The Journal of allergy and clinical immunology.

[101] Langmead B, Salzberg SL (2012). Fast gapped-read alignment with Bowtie 2. Nature methods.

[102] Anders S, Huber W (2010). Differential expression analysis for sequence count data. Genome Biol.

[103] Xia J, Gill EE, Hancock RE (2015). NetworkAnalyst for statistical, visual and network-based meta-analysis of gene expression data. Nat Protoc.

[104] Xia J, Benner MJ, Hancock RE (2014). NetworkAnalyst–integrative approaches for protein-protein interaction network analysis and visual exploration. Nucleic acids research.

[105] Akalin A (2012). methylKit: a comprehensive R package for the analysis of genome-wide DNA methylation profiles. Genome biology.

[106] Wang HQ, Tuominen LK, Tsai CJ (2011). SLIM: a sliding linear model for estimating the proportion of true null hypotheses in datasets with dependence structures. Bioinformatics.

