# Supplementary material for: TET1 contributes to allergic airway inflammation and regulates interferon and aryl hydrocarbon receptor signaling pathways in bronchial epithelial cells
Source: Sci Rep. 2019 May 14;9:7361. doi: 10.1038/s41598-019-43767-6 (PMC6517446; doi:10.1038/s41598-019-43767-6)
Supplement: Supplementary file 1 — Supplementary Information [file 41598_2019_43767_MOESM1_ESM.pdf]

TET1 contributes to allergic airway inflammation and regulates interferon and aryl hydrocarbon receptor signaling pathways in bronchial epithelial cells.

JD Burleson, BS, [1], Dylan Siniard, MS, [1,2], Veda K. Yadagiri, MS, [1], Xiaoting Chen, PhD, [3], Matthew T. Weirauch, PhD, [3,4,5], Brandy P. Ruff, BS, [1], Eric B. Brandt, PhD, [1], Gurjit K. Khurana Hershey, MD, PhD, [1,5], Hong Ji, PhD,[1,2,5,6,7]*

[1]Division of Asthma Research; [2]Pyrosequencing lab for genomic and epigenomic research; Cincinnati Children's Hospital Medical Center, Cincinnati, OH, USA; [3]Center for Autoimmune Genomics and Etiology, Cincinnati Children's Hospital Medical Center, Cincinnati, OH, USA; [4]Divisions of Biomedical Informatics and Developmental Biology, Cincinnati Children's Hospital Medical Center, Cincinnati, OH, USA; [5]Department of Pediatrics, University of Cincinnati College of Medicine, Cincinnati, OH, USA; [6]Department of Anatomy, Physiology and Cell Biology, School of Veterinary Medicine, University of California, Davis, CA, USA; [7]California National Primate Research Center, USA.

***Corresponding author**: Hong Ji, PhD, Department of Anatomy, Physiology and Cell Biology, School of Veterinary Medicine, University of California, Davis, CA, USA; California National Primate Research Center, USA. Phone: 530-754-0679. Email: hgji@ucdavis.edu

Supplementary Table S9. Annotations of CG sites in Irf7 gene presented in Figure 7 and primers used for pyrosequencing.

Name	Genome Location	Strand	Position	Pyro Assay
CG1	Chr7:141269462	+	Upstream TSS	N/A
CG2	Chr7:141269419	+	Upstream TSS	N/A
CG3	Chr7:141266201	-	Gene body	N/A
CG4	Chr7:141264224	-	Gene body	F primer: 5'-TGGTAGGTGGAAGTTGTTTTTAGTT-3'<br>R primer: 5'-biotin-ACATTTTATCCCATACCCCCTAC-3'<br>S primer: 5-GTTTTTAGTTTTAGGTTTTAATAG-3'
CG5	Chr7:141264202	-	Gene body	

Supplementary Table S11. RT-qPCR primers

Gene	Forward	Reverse
**Human**		
**hTET1**	5'-CCCGGGCTCAAAGTTGTG-3'	5'-GCAGGAAACAGAGTCATTGGTCCT-3'
**hIRF7**	5'-CCCCATCTTCGACTTCAGAG -3'	5'-AAGGAAGCACTCGATGTCGT -3'
**hIFNα1**	5'-CAGAGTCACCCATCTCAGCA -3'	5'-CACCACCAGGACCATCAGTA -3'
**hIFNα2**	5'-CTGGCACAAATGGGAAGAAT-3'	5'-CTTGAGCCTTCTGGAACTGG -3'
**hIFNβ**	5'-AAACTCATGAGCAGTCTGCA -3'	5'-AGGAGATCTTCAGTTTCGGAGG -3'
**GAPDH**	5'-GGGGAAGGTGAAGGTCGGAGTCA-3'	5'-AGCCTTGACGGTGCCATGGAAT-3'
**Mouse**		
**mMuc5ac**	5'-CCATGAAGTGGGAGTGTGTG-3'	5'-TTGGGATAGCATCCTTCCAG-3'
**mMuc5b**	5'-CTCGTGTACAAACGGGAAGT-3'	5'-TGTGCTGAAACATTCCACAT-3'
**mIl13**	5'-AGACTCCCCTGTGCAACGGCA-3'	5'-GGAGACCGTAGTGGGGGCCTT-3'
**mIl4**	5'-CTGACGGCACAGAGCTATTGA-3'	5'-TATGCGAAGCACCTTGGAAGC-3'
**mTslp**	5'-TCAATCCTATCCCTGGCTG-3'	5'-GCATGAAGGAATACCACAATCTTA-3'
**mIl33**	5'-TCCCAACAGAAGACCAAAG-3'	5'- GATACTGCCAAGCAAGGAT-3'
**mIL17a**	5'-CAGCAGCGATCATCCCTCAAAG-3'	5'-CAGGACCAGGATCTCTTGCTG-3'
**mIL17f**	5'-CTGGAGGATAACACTGTGAGAGT-3'	5'-TGCTGAATGGCGACGGAGTTC-3'
**mIl6**	5'-GCTCTTGCTTGCCTTG-3'	5'-GCTGGAGACCGTAGTG-3'
**mIl1β**	5'-AAGCCTCGTGCTGTCGGACC-3'	5'-CCAGCTGCAGGGTGGGTGTG-3'
**mTgfα**	5'-GCTACTCGCCAACCGCAGGG-3'	5'-ACCCAGAGTGGCAGACACATGC-3'
**mEgfr**	5'-GCGTGGAGAAATGCAACATC-3'	5'-ATTCTGGATGGCACTGGATG-3'
**mTff2**	5'-TTCCAAACCAAGAATCGGAG-3'	5'-CTCGGCAGTAGCAACTCTCA-3'
**mRpl13a**	5'-ATGACAAGAAAAAGCGGATG-3'	5'-CTTTTCTGCCTGTTTCCGTA3'

Supplementary Figure S1. Tet1 regulates genes in IFN and AhR signaling pathways in human bronchial epithelial cells. a. Activation of endogenous *TET1* locus by recruitment of histone deacetylase increased *TET1* expression. Expression values of four samples (including two RT-qPCR technical replicates from two technical transfection replicates) measure by RT-qPCR were normalized to the expression of GADPH. Mean±SEM for each group is shown. b. Suppression of *IRF7* expression by *TET1* activation. RPKM from RNA-seq analysis on two

biologic replicates was plotted. c. Top 11 significantly enriched pathways from IPA analysis. d. Protein-protein interaction network (1st order subnetwork). In c and d, green color marks gene with downregulated expression in the input dataset, red color marks genes with upregulated expression. Genes involved in IFN signaling are highlighted by blue circles.

Supplementary Figure S2. Expression of *Tet1*, *Dnmt1* and *Dnmt3a* in the lungs of mice with or without HDM challenges. A. *Tet1*. B. *Dnmt1*. C. *Dnmt3a*. Expression values were measured by RT-qPCR and normalized to the expression of mRPL13A**.** $Tet1^{+/+}$ Saline, n=7-8; $Tet1^{+/+}$ HDM, n=6-7; $Tet1^{-/-}$ Saline, n=5-7; $Tet1^{-/-}$ HDM, n=7. Mean±SEM for each group is shown. Data are normally distributed and student t-tests were applied with Bonferroni corrections for multiple testing. *p<0.05, **p<0.01, ***p<0.001, ****p<0.0001. ns represents not significant.

Supplementary Figure S3. Loss of TET1 have no impact on extracellular free DNA produced by the lungs following HDM challenges. dsDNA in BALF from indicated mice were measured. $Tet1^{+/+}$ Saline, n=5; $Tet1^{+/+}$ HDM, n=5; $Tet1^{+/-}$ Saline, n=6, $Tet1^{+/-}$ HDM, n=6; $Tet1^{-/-}$ Saline, n=3; $Tet1^{-/-}$ HDM, n=6. Mean±SEM for each group is shown. Data are normally distributed and unpaired student t-tests were applied with Bonferroni corrections for multiple testing. *p<0.05, **p<0.01.

a.

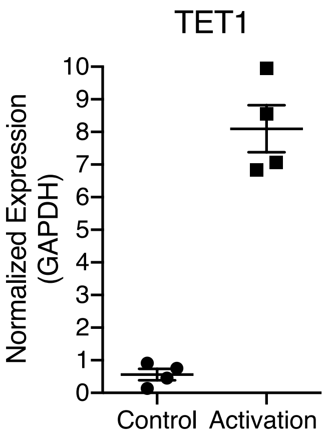

b.

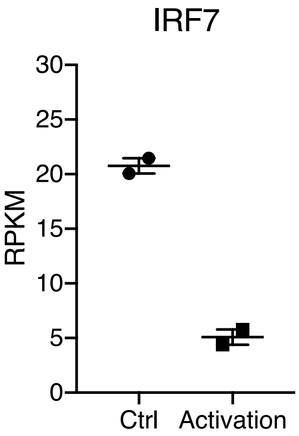

c. Top enriched pathways

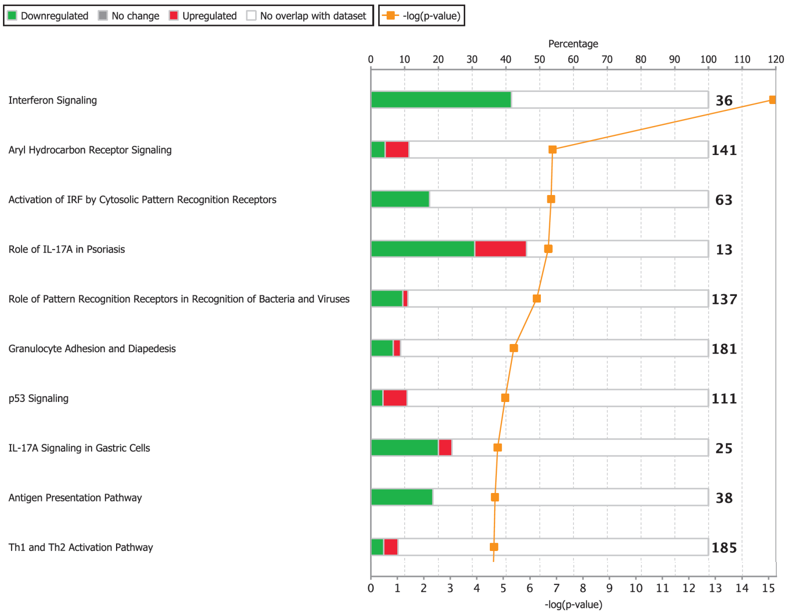

d. Protein-protein interaction network:

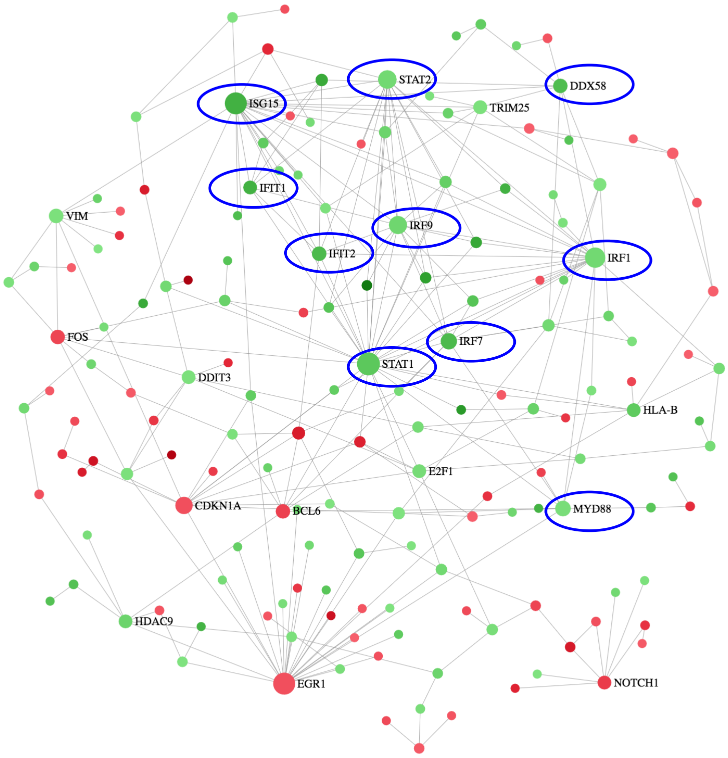

Supplementary Figure S1

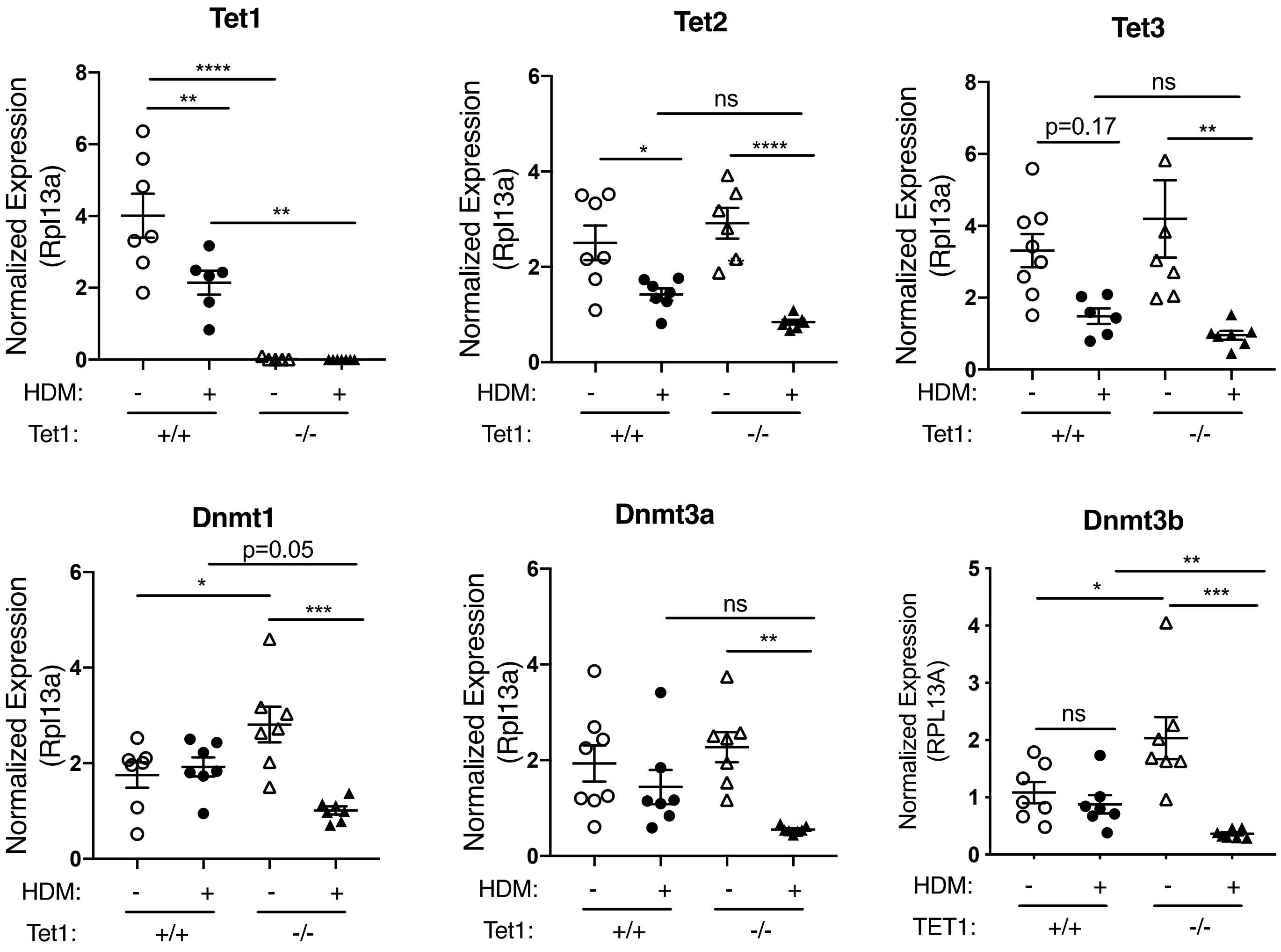

Supplementary Figure S2

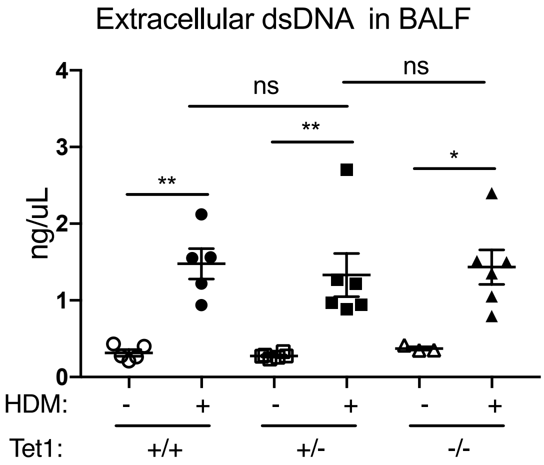

Supplementary Figure S3
